# Notch Signaling in Pancreatic Development

**DOI:** 10.3390/ijms17010048

**Published:** 2015-12-30

**Authors:** Xu-Yan Li, Wen-Jun Zhai, Chun-Bo Teng

**Affiliations:** 1 College of Life Science, Northeast Forestry University, Harbin 150040, China; lxy0702@126.com (X.-Y.L.); makawada@163.com (W.-J.Z.); 2 College of Life Sciences, Agriculture and Forestry, Qiqihar University, Qiqihar 161006, China

**Keywords:** pancreatic progenitor, Notch signaling, differentiation, maintenance

## Abstract

The Notch signaling pathway plays a significant role in embryonic cell fate determination and adult tissue homeostasis. Various studies have demonstrated the deep involvement of Notch signaling in the development of the pancreas and the lateral inhibition of Notch signaling in pancreatic progenitor differentiation and maintenance. The targeted inactivation of the Notch pathway components promotes premature differentiation of the endocrine pancreas. However, there is still the contrary opinion that Notch signaling specifies the endocrine lineage. Here, we review the current knowledge of the Notch signaling pathway in pancreatic development and its crosstalk with the Wingless and INT-1 (Wnt) and fibroblast growth factor (FGF) pathways.

## 1. Introduction

Notch signaling is an evolutionarily conserved pathway for cell-cell communication and cell-fate determination during embryonic development and tissue homeostasis [1,2]; it includes canonical and non-canonical pathways. The former is initiated by ligand-receptor interactions between adjacent cells [3,4], which results in the activation of the *Hes1* gene by a complex consisting of the Notch intracellular domain (NICD), Rbp-Jκ family of nuclear proteins (Rbp-J (mammalian), Su(H) (*Drosophila*), and Lag-1 (*Caenorhabditis elegans*)), and the Mastermind co-activator. However, non-canonical Notch signaling broadly encompasses several modes of Notch activity that do not go through the Rbp-J and activation of the *Hes/Hey* genes [5,6]. Here, we review the role of the canonical pathway during pancreatic development. Notch-mediated lateral inhibition represents an important conserved mechanism that regulates cell differentiation, cell proliferation and cell survival in stem cells [7,8,9]. Abnormalities in Notch signaling have been linked to various syndromes and diseases, including developmental malformation, neurodegenerative diseases, metabolic disorders, and malignant disease [10,11,12,13].

Over the past two decades, numerous reports have revealed the pivotal role of Notch signaling in pancreatic specification, cell proliferation, differentiation and plasticity [9,14,15,16]. The first evidence of the involvement of the Notch signaling pathway in pancreatic development focused on its lateral inhibition role in controlling pancreatic fate decision. The activation of Notch signaling in pancreatic progenitors prevents their differentiation into the endocrine or exocrine cell lineage [17,18]. In contrast, the blockage of the Notch signaling pathway causes premature differentiation of the multipotent progenitor cells (MPCs) into endocrine cells [19,20]. A series of studies have revealed that Notch signaling functions as a negative regulator of the pro-endocrine factor neurogenin3 (*Ngn3*), and the formation of insulin-producing β-cells is significantly enhanced by induction of pro-endocrine factors or inhibition of Notch processing. However, recently, some researchers have disagreed with the notion that the Notch pathway is an inhibitor of endocrine cell differentiation. They have proposed that the Notch pathway specifies the pancreatic progenitors differentiating towards endocrine lineage [21,22] or the inactivated Notch pathway promotes acinar cell differentiation [23,24,25]. Some recent findings have revealed that Notch signaling does not act through an on or off mode, but at a Notch level-dependent manner to regulate the quiescence, self-renewal and differentiation of pancreatic progenitor cells during pancreas development [22,26]. Studies on animal models of pancreatic regeneration and diseases have revealed that Notch signaling is involved in controlling the plasticity of terminally differentiated adult pancreatic cells [25,27,28]. During pancreatic development, the formation of islets of Langerhans is enhanced by specific transcription factors and regulated by multiple intercellular signaling pathways, including the Wingless and INT-1 (Wnt), fibroblast growth factor (FGF), Notch pathways, *etc.* [29,30,31]. These pathways independently or collaboratively perform regulatory functions at different time-points. This mini review summarizes the current knowledge of the roles of Notch signaling in pancreatic development, including pancreatic cell lineage commitment, pancreatic progenitor maintenance, and adult pancreatic cell plasticity, and it also discusses the crosstalk between the Notch and Wnt/FGF pathways.

## 2. An Outline of Pancreatic Development

**Figure 1:**
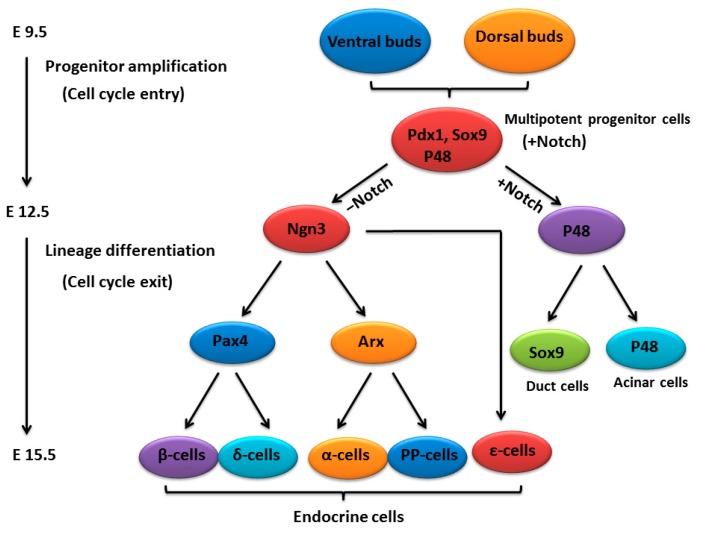
A schematic of pancreatic development.

The mammalian pancreas is derived from two independent ventral and dorsal buds and experiences three stages of transition [32,33]. In mice, the primary transition is marked by the specification and proliferation of pancreatic progenitors and the appearance of glucagon-producing cells during E9.5 and E12.5 [34,35]. The secondary transition is from E13.5 to E15.5, during which all five hormone-expressing endocrine lineages (α-, β-, δ-, ε-, and PP-cells) begin to emerge rapidly and amylase-expressing acinar cells arise from the extending tip epithelium [36,37]. The third transition occurs from E16.5 to E19. During this period, endocrine cells migrate and cluster into numerous islets, and acinar cells further expand [38]. The pancreatic buds contain undifferentiated progenitor cells, which contribute to all pancreatic cell lineages, the exocrine, ductal and endocrine cell lineages [39,40]. In the MPCs, Notch signaling is critical and essential for their proliferation and commitment [22,24] (Figure 1).

Lineage-specific transcription factors control the differentiation of pancreatic progenitor cells towards a specific type [40,41]. Pancreatic cells arise from pancreatic duodenal homeobox 1 (Pdx1)-expressing progenitors (Figure 1). As pancreatic development proceeds, the *Pdx1* gene becomes progressively confined to endocrine β-cells, where it plays an essential role in the transcriptional activation of the *insulin* gene [42]. The progenitors co-express sex-determining region Y (Sry) box 9 (Sox9) and pancreas specific transcription factor1a (Ptf1a, also known as P48) [43,44] (Figure 1). However, Sox9 expression is eventually limited to a subset of ductal and centroacinar cells (CACs) in adults, and Ptf1a is expressed solely in mature acinar cells [45,46] (Figure 1). The Basic Helix-Loop-Helix (bHLH) transcription factor Ngn3 drives MPCs towards the endocrine cell fate [47]. The specification of the endocrine subtypes is essentially under the control of the opposing actions of aristaless related homeobox (Arx) and paired box 4 (Pax4) acting downstream of Ngn3 [48] (Figure 1). *Ngn3*-null mice fail to generate pancreatic endocrine cells and lose the expression of islet transcription factors, including islet-1 (*Isl1*), NK2 homeobox 2 (*Nkx2.2*), paired box 4 (*Pax4*), paired box 6 (*Pax6*) and *NeuroD1*, which are all important for endocrine cell differentiation [49]. During the maturation of islet cells, the cell cycle regulatory proteins play a pivotal role in cell division and differentiation. Prior to and during the secondary transition, cyclin-dependent kinase 4 (Cdk4) and E2F transcription factor 1 (E2F1) promote β-cell development by activating Ngn3 to increase the numbers of endocrine precursors [50]. In the mouse embryonic pancreas, P21 protein (Cdc42/Rac)-activated kinase 3 (Pak3) acts downstream of Ngn3 to promote cell cycle exit and cell differentiation by repressing *cyclin D1* in Ngn3^+^ endocrine progenitors [51]. The PTF1 complex initiates exocrine differentiation [52]. The PTF1 complex, which is composed of p64/HEB, p75/E2A and Ptf1a [52], directly binds to the promoter regions of the acinar digestive enzyme genes, and leads to acinar cell differentiation and cell cycle exit [46,53]. More interestingly, a recent study on the *Ptf1a* mutant zebrafish model has found that the down-regulation of Ptf1a induces acinar-to-endocrine fate conversion [54]. Using genetic loss- and gain-of-function approaches, Schaffer *et al.* [55], have demonstrated that the cross-repressive interactions between Nkx6 (Nkx6.1/Nkx6.2) and Ptf1a commit the fate of pancreatic progenitor cells. Nkx6 induces endocrine cell determination, however, Ptf1a promotes acinar cell specification.The cross-antagonistic switch between Nkx6 and Ptf1a is controlled by Notch signaling [55].

Growing evidence suggests that miRNAs play an important role in the embryonic development and physiological function of pancreas. Examples of these miRNAs include miR-7, miR-375, the miR17-92 cluster, miR-26 and miR-15 *et al.* [56]. *Dicer*-null mice display gross defects in all pancreatic lineages, especially the insulin-producing-cells. The endocrine defect in *Dicer*-null mice is associated with an increase in the expression of the Notch signaling target Hes1 [56]. miR-7 is dispensable for the maintenance of β-cell mass and functions [57]. miR-375 is required for normal pancreatic genesis and maintains α- and β-cell mass [58]. We have found that, during pancreatic progenitor cell differentiation, miR-375 inhibited pancreatic progenitor cell proliferation by targeting the Hippo signaling effector *Yap1* [59]. miR-375 also plays a role in regulating insulin secretion through targeting the myotrophin (*Mtpn*) and pyruvate dehydrogenase kinase (*Pdk1*) genes. miR-19b and miR-18a have been shown to directly act on *NeuroD1* and *Ptf1a* 3′ UTR, respectively [60,61]. These two miRNAs may contribute to the regulation of the differentiation and function of β-cells and acinar cells during pancreatic development. Pancreatic regeneration is accompanied by the high expression of miR-15a, miR-15b, miR-16 and miR-195, which can potentially bind to the *Ngn3* transcript [62].

The development of the pancreas depends on the spatio-temporal expression of a number of transcription factors and associated signaling pathways [63,64]. Studies on the transcriptional regulation of the pancreas have been reviewed extensively, and thus, not discussed in detail here.

## 3. An Overview of the Notch Signaling Cascade

**Figure 2:**
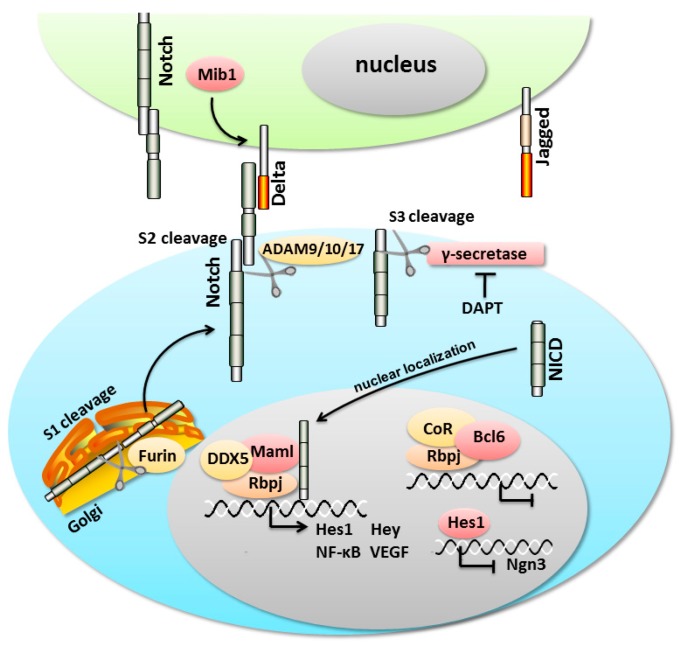
A schematic of Notch signaling during pancreatic development. Notch signaling is initiated by the binding of the ligands and receptors of two neighboring cells. Upon activation, Notch is cleaved, releasing the Notch intracellular domain (NICD). The NICD can subsequently translocate into the nucleus to transcriptionally activate Notch target genes. Hes1 inhibits the expression of *Ngn3* by blocking its promoter activity.

Notch genes encode a single-pass transmembrane receptor family of molecules, including Notch1–4 in mammals [65]. Notch proteins contain a large Notch extracellular domain (NECD) composed of 29–36 tandem epidermal growth factor-like repeats, a short Notch transmembrane fragment (NTM), and a Notch intracellular domain (NICD) [66]. As part of the biosynthetic process of Notch, a Furin-like protease in the Golgi cuts nascent Notch proteins at the S1 cleavage site into two fragments [67,68], the NECD and non-NECD domains. Then, they conjugate non-covalently as a heterodimer and target to the cellular surface. The initiation of Notch signaling is trigged by the binding of the NECD domain to type I transmembrane ligands (δ-like 1, 3, 4 (Dll1, 3, 4) and Serrate/Jagged1, 2 (Ser/Jag1, 2)) on adjacent cells. Then, the membrane-bound proteases are activated and continue to cleave the non-NECD domain into two domains, the NTM and NICD domains. This cleavage is catalyzed by A Disintegrin and Metalloprotease (ADAM)-family of metalloproteases at a luminal juxtamembrane site 2 (S2) and by a tetrameric γ-secretase complex at an intramembrane site 3 (S3) of non-NECD domain. After the transcriptionally activated NICD is released and translocates into the nucleus, it binds to Rbp-J [69]. Then, Rbp-J recruits the coactivator Mastermind-like (Maml) to activate downstream genes, including the Hairy enhance of split (*Hes*) and Hairy/enhancer of spit related with YRPW motif (*Hey*) families, nuclear factor-κB (*NF-κB*), vascular growth factor receptor (*VEGF*), *etc.* [70,71] (Figure 2). The *Hes* genes, which encode bHLH transcriptional repressors, play crucial roles in the fate choice and differentiation of stem cells, e.g., in the inner ear, neurons and pancreas development [72,73,74]. Activated Hes1 inhibits the expression of *Ngn3* by binding to the proximal promoter and specifically blocking promoter activity [75,76]. However, when Notch signaling is limited or nonexistent, Rbp-J recruits a corepressor (CoR) complex to repress the expression of the Notch target genes (Figure 2).

Several Notch-related regulators have also been found to function in normal organism development and tumorigenesis. In the left-right asymmetric development of *Xenopus*, the transcriptional repressor B cell leukemia/lymphoma 6 (Bcl6) competes with Maml to repress the Notch1 target genes [77]. Mind bomb1 (Mib1), which encodes an E3 ubiquitin ligase, promotes the internalization of Δ, which paradoxically increases the efficiency with which Δ activates Notch [78]. It is reported that Mib1 is required for the formation of pancreatic β-cells and the specification of neurons and glia in the spinal cord [79,80]. In neural progenitors, the multifunctional protein kinase Nemo-like kinase (NLK) negatively regulates the formation of the Rbp-J-NICD-Maml complex through the phosphorylation of Notch1 to fine-tune the timing of neuronal differentiation [81]. DEAD (Asp-Glu-Ala-Asp) box helicase5 (DDX5, also known as p68) is found to act as a component of the Mastermind-like1(Maml1) protein complex for transcriptional coactivation and is also a novel regulator of oncogenic Notch signaling in T-ALL leukemic cells [82]. Several reports have shown that the Notch glycosylating enzyme Manic Fringe (Mfng) is expressed in Ngn3^+^ cells, and the ectopic expression of Mfng is sufficient to induce chick endodermal cells to differentiate towards endocrine fate [83]. However, the deletion of *Mfng* in mice has no effect on pancreatic development, cell differentiation or function [84]. The glycosylation of the Notch receptors has been shown to prevent the binding of Jagged, thus inhibiting Jagged-mediated activation [85,86]. The ADAM family of metalloproteases have proteolytic activity and play a major role in the ectodomain shedding of proteins involved in paracrine signaling, cell adhesion and intracellular signaling [87,88,89]. During embryogenesis, ADAM9 and ADAM17 are expressed separately in insulin-producing β-cells and other islet cells, and ADAM10 is predominantly detectable in acinar cells [90]. ADAM10 and ADAM17 have been shown to cleave Notch at the S2 site, and ADAM9 and ADAM15 are also found to be involved in Notch signaling indirectly in neurons [91,92]. These ADAMs may play an essential role in the activation of Notch signaling during pancreatic development.

## 4. Notch Signaling Pathway in the Pancreas

### 4.1. Notch Signaling in Pancreatic Progenitor Cell Differentiation

Notch signaling is critical at multiple steps during pancreatic development [16,93]. The expression levels of the Notch ligand and receptor change during pancreatic development. Notch1 is the first expressed receptor in a subset of pancreatic epithelium cells at E9.5 and then is broadly expressed in the pancreatic epithelium at E14.5 [94]. Notch2 is strongly expressed at E11.5, and its expression is absolutely restricted to ductal cells at E15.5 [94]. Notch3 and Notch4 are expressed in the early pancreatic mesenchyme and then in the endothelial cells of the pancreas at E15.5 [94]. Dll1 is transiently expressed in the pancreatic duct epithelium between E9.5 and E11.5 [93,95], and Jag1 is the most abundant ligand during mid-gestation pancreatic development [96]. Recent studies have identified that CACs and terminal duct cells are unique locations of activated Notch signaling in the pancreas of adult mice and zebrafish [97,98]. In the adult pancreas, the reactivation of Notch is involved in phenotype modulation of adult rat exocrine cells and the proliferation of metaplastic exocrine cells during pancreas regeneration [99,100]. In the caerulein-induced pancreatitis mouse model, the expression of the exocrine genes disappeared in pancreatic exocrine cells, while genes normally associated with Notch components, such as *Notch1*, *Notch2*, *Hes1*, *Jag2*, and a low level of *Dll1* were induced [101].

**Figure 3:**
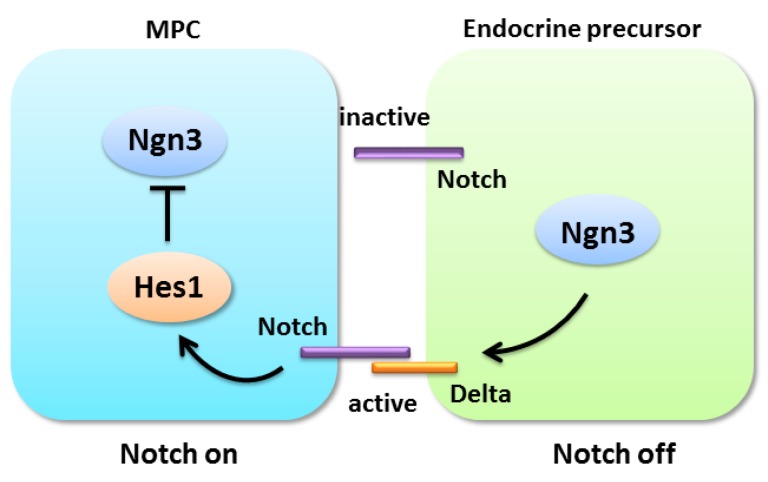
Notch-mediated lateral inhibition in pancreatic multipotent progenitor cell differentiation.

During the secondary transition, Notch regulates endocrine differentiation via a lateral inhibition mechanism. The lateral inhibition model proposes that the onset of Ngn3 expression initiates endocrine differentiation and activates the Notch ligand Delta. Subsequently, Delta binds with Notch receptors in neighboring cells to initiate the Notch signaling cascade and release the NICD. The activated NICD enters the nucleus to activate the target gene *Hes1*, which inhibits the expression of Ngn3 [75,76]. Ultimately, the activated Notch pathway prevents adjacent cells from adopting an endocrine fate (Figure 3). Most early studies have shown that the inactivation of Notch signaling accelerates the premature differentiation of the endocrine pancreas [20]. In *Dll1*-deficient mice, Pdx1^+^ progenitor cells within the pancreatic buds lacked expansion and differentiated prematurely into endocrine cells [93]. In zebrafish embryos, the inhibition of *Jagged* caused ectopic islet-cell differentiation [19]. The loss of *Rbp-J* at the initial stage of pancreatic development resulted in the rapid differentiation of α-and PP-cells and decreased numbers of Ngn3^+^ cells [102]. Furthermore, *Rbp-J* KO mice exhibited insulin-deficient diabetes [20,102]. *Hes1*-deficient mice showed pancreatic hypoplasia, which is caused by the depletion of pancreatic epithelial progenitors from the accelerated differentiation of pancreatic endocrine cells [72,103]. A similar phenotype has been observed in mice that over-express *Ngn3* [104] or the intracellular form of *Notch3* (a repressor of Notch signaling) [105]. Conversely, the enforced activation of Notch signaling blocks pancreatic progenitors differentiation into the endocrine and exocrine cells. In chicken embryos, activated *Notch1* can block the expression of endocrine genes and prevent endocrine differentiation [18]. The lentiviral-mediated activation of *Hes1* and intracellular domain of Notch (*Notch-IC*) in mouse dorsal pancreatic buds at E10.5 represses endocrine and exocrine differentiation [106]. Ectopic *Notch* activation in zebrafish embryos can inhibit acinar and β-cell differentiation [106]. When *Notch* was overexpressed in the E9.5 or E11.5 mouse embryonic pancreas, the differentiation of Hes1^+^ cells was blocked; however, ectopic *Notch* activation at E15.5 does not perturb exocrine differentiation [107]. The sustained Notch1 signaling in Pdx1-NICD transgenic mice prevents the differentiation of pancreatic acinar and endocrine cells [17].

Furthermore, recent studies have implied that the Notch-mediated regulation of endocrine differentiation is more complex than the classical lateral inhibition model suggests. For example, E9.5 *Dll1* mutant mice show a dramatic decrease in their number of Ngn3^+^ cells, which suggests that Dll1 is required for the continuous formation of Ngn3^+^ endocrine precursors [21]. Shih *et al.* [22], demonstrated that Notch induces the expression of the *Ngn3* gene activator Sox9, which may provide further evidence that Notch initiates the endocrine lineage. Some current gene-targeting studies focused on the Notch signaling components found that inactivated Notch signaling promotes the differentiation or transdifferentiation of MPCs, CACs or adult duct cells to the acinar lineage, which suggests that Notch may specify the endocrine lineage. Zebrafish embryos that were injected with RNA encoding a dominant-negative Suppressor of Hairless showed accelerated exocrine cells compared with the controls [106]. Similarly, a study by Cras and his colleagues demonstrated that the deletion of presenilin1 (*Psen1*) and presenilin2 (*Psen2*) drives Ngn3-expressing cells to differentiate into acinar cells [108]. Once cultured in the presence of the γ-secretase inhibitor DAPT, mouse pancreatic progenitors differentiate into acinar cells [23]. When Notch signaling is suppressed through the mosaic overexpression of a Notch signaling antagonist, dominant-negative mastermind-like1 (dnMaml), the pancreatic progenitor cells subsequently differentiate into acinar cells [24]. The inactivation of *Mib1* in the endoderm causes a loss of endocrine cells and an increase in exocrine cells in the proximal domain, which suggested that Notch signalling is required to prompt MPCs to adopt an endocrine fate [79]. Upon *Rbp-J* deletion, mouse CACs undergo a rapid conversion to acinar cells [109]. In addition, the differentiation of acinar cells is accelerated by *Hes1* depletion but is suppressed by NICD induction in adult mouse Sox9-expressing cells [25].

The causal role of Notch signaling in the patterning of MPCs into endocrine cells has been evaluated in some studies in different aspects. Genetic interactions have suggested that γ-secretase and Notch2 act in a non-canonical mechanism to sequester Rbp-J away from Ptf1a, which secures mouse Ngn3-positive progenitor cells to the endocrine fate [108]. Afelik *et al.* [24], found that Notch signaling is required to establish a duct and endocrine identity through the activation of Nkx6.1, which is bound to Rbp-J. Some studies revealed that Notch does not function in an on-off mode, but that Notch signaling seems to act in a concentration-dependent manner [22,26,76]. A study by Shih *et al.* [22], proposed a model in which high Notch expression activates Hes1 and Sox9 expression, resulting in the generation of pancreatic duct cells, while low Notch expression activates Sox9 but not Hes1, resulting in Ngn3 activation and endocrine differentiation. A study on the dynamic assessment of Notch signaling in the zebrafish intrapancreatic duct found that the proliferation and differentiation of MPCs are regulated by different levels of Notch signaling [26]. It has been reported that the hyperactivation of Notch signaling could convert the proliferative Notch-responsive MPCs to the quiescent state, hypo-activation of the Notch pathway induces the quiescent MPCs to the proliferative state, and strong down-regulation of Notch signaling promotes MPCs differentiate towards endocrine cells [26]. However, there are gaps in our understanding of the timing and extent of Notch ligand-receptor interactions and how this affects the behavior of MPCs. Therefore, there may be even more complexity in the Notch-mediated regulation of pancreatic development.

### 4.2. Notch Signaling in Pancreatic Progenitors Maintenance

The Notch signaling pathway has been found to maintain proliferation and prevent the precocious differentiation of pancreatic progenitor cells [18,110]. The activation of Notch at the “primary transition” maintains the pancreatic state, allowing the coordination of epithelial outgrowth and helping the pancreatic buds reach their destined size.

**Figure 4:**
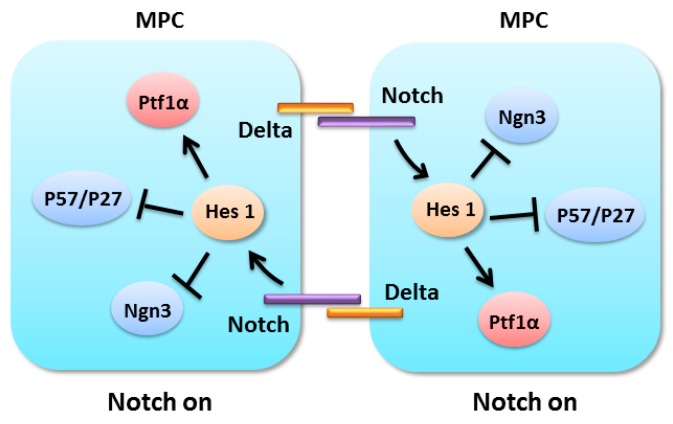
Notch signaling in the proliferation of pancreatic multipotent progenitor cells.

The transcription factor network is crucial for the maintenance and expansion of MPCs. Pdx1 and Ptf1a can form autoregulatory loops and feed-forward loop to retain and expand the progenitor cells during early pancreatic development [111]. The inactivation of Ptf1a in mice results in pancreatic hypoplasia, glucose intolerance and the transformation of pancreatic progenitors to a duodenal fate [112,113]. The expression of Pdx1 and Ptf1a is also regulated by fibroblast growth factor 10 (FGF10) and Notch signaling [111]. FGF10 signaling promotes the expansion of pancreatic epithelial cells through Sox9 and Hes1 activation [31,110,114,115]. Hes1 is reported to regulate the binary decision choice of pancreatic progenitors, cell cycle exist or self-renewal maintenance, through suppression of P57 and P27, which are cyclin-dependent kinase inhibitors [116,117] (Figure 4). In pancreatic progenitors, the inactivation of Hes1 could increase the expression of the *P57* gene, which leads to cell cycle arrest, early differentiation and the depletion of the progenitor pool [117]. Moreover, Sox9 promotes pancreatic progenitor expansion by modulating the FGF-receptor (FGFR), Notch and Wnt signal transduction [31,45,118]. It has been reported that the pancreas-specific *Sox9*-deficient progenitors exhibit reduced proliferation and a low level of Hes1 [45]. Sox9 cell-autonomously controls the expression of FGFR2b in pancreatic progenitors [31]. In isolated human islet-epithelial clusters, the knockdown of *Sox9* resulted in a decrease in pGSK3β, nuclear β-catenin and the Wnt signaling target gene *cyclin D1* [118]. Notch signaling also regulates Sox9 expression in the pancreas [22,25]. Notch positively regulates Sox9 expression in a Hes1-independent manner in the pancreatic duct cells of *Sox9^CreERT2^* mice [25]. The progenitor-intrinsic transcription factors Pdx1, Ptf1a and Sox9, as well as Notch, Wnt and FGF10 signaling, compose a complex network for the maintenance of MPCs.

### 4.3. Notch Signaling in Adult Pancreatic Cell Plasticity

Because the components of the Notch signaling pathway are not normally expressed in terminally differentiated pancreatic cells, most mature cells lose their responsiveness to Notch signaling. However, in-depth studies have indicated that Notch signaling is involved in controlling the plasticity of adult, terminally differentiated pancreatic cells. Importantly, the dedifferentiation and transdifferentiation of terminally differentiated pancreatic cells are associated with Notch pathway reactivation during regeneration following pancreatitis [28,99,119], pancreatic neoplasia [120], and acino-ductal metaplasia [121,122]. For instance, when the pancreas is injured by pancreatitis, the exocrine acinar cells lose their differentiated characteristics and present acino-ductal metaplasia, which strongly upregulates the expression of the receptors Notch1 and 2 and the target genes *Hes1*, *Hey1*, and *Hey2* [27]. Adult pancreatic duct cells retain their plasticity to differentiate into endocrine or acinar cell types, which is controlled by Notch signaling and Sox9 cooperatively [25]. When dissociated adult human β-cells are cultured in serum-containing medium, the β-cells dedifferentiate, and they enter into the cell cycle, which correlates with the activation of the Notch pathway and the down-regulation of P57 [123]. When Hes1 expression was down-regulated by shRNA, the dedifferentiated β cells redifferentiate into insulin-expressing cells [124]. Notch signaling is also reported as a gatekeeper of acinar-to-β-cell conversion *in vitro* [100,125]. Baeyens *et al.* [100], found that the growth factor-induced conversion of adult acinar cells to β-cells is negatively regulated by activated Notch1, which has the ability to prevent the re-expression of the pro-endocrine transcription factor Ngn3. Notch re-expression is deeply associated with the modulation of the proliferation of metaplastic cells and possible plays an important role in pancreatic regeneration.

## 5. The Crosstalk between Notch Signaling and the Wingless and INT-1 (Wnt)/Fibroblast Growth Factor (FGF) Pathway in the Pancreas

### 5.1. Notch/Wnt Crosstalk

Notch and Wnt signaling are key pathways that control the expansion and differentiation of stem/progenitor cells during embryogenesis, tissue formation and maintenance in adult homeostasis [126]. The Wnt pathway inhibits the specification of the pancreas in the early endoderm, whereas the pathway promotes the growth of the dorsal and ventral primordial buds, specifically the proliferation of acinar cells [127]. In the developing mouse pancreatic epithelium, the deletion of *Wnt7b* leads to pancreatic hypoplasia because of the reduced proliferation of pancreatic progenitor cells [128]. The ectopic stabilization of *β-catenin* before E11.5 in mouse embryos prevents the proper differentiation and expansion of early pancreatic progenitor cells [129]. Ectopic expression of *β-catenin* in mouse embryos at E18.5 causes the gross enlargement of the exocrine pancreas, which results in a dramatic increase in pancreas organ size [129]. Conversely, in pancreas-specific *β-catenin* knockout mice, the pancreas almost completely lacks acinar cells [130]. Meanwhile, gain- and loss-of-function experiments have proved that the Wnt pathway is involved in β-cell growth and survival. The proliferation of mouse islet cells and β-cells increases when they are treated with Wnt3a [131,132]. Conditional activated β-catenin promotes the expansion of β-cells in mice [129]. However, the addition of the soluble Wnt inhibitor Fz8-cysteine-rich domain (Fz8-CRD) or the conditional knock-in of the Wnt inhibitor Axin impaired the proliferation of neonatal mouse β-cells [129,131]. Likewise, studies have found that the Wnt signaling pathway plays a role in regulating glucose-stimulated insulin secretion in mature β-cells and is involved in lipid metabolism and glucose homeostasis [133,134]. The knockout of the low-density lipoprotein receptor-related protein 5 (LRP5), which is a Wnt co-receptor, results in glucose intolerance in mice [133].

**Figure 5:**
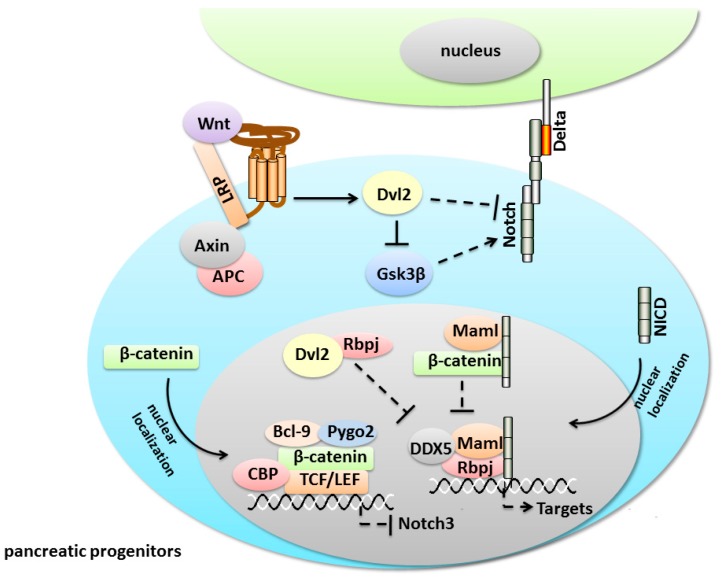
Inhibitory crosstalk between the Wingless and INT-1 (Wnt) and Notch pathways in pancreatic progenitor cells. The dashed lines signify that the mechanisms existing in the pancreas are unknown. The arrows represent activation whereas bar-headed lines represent inhibition.

In the early pancreatic lineage commitment, there is a repressive crosstalk between Notch and Wnt signaling. Notch pathway promotes the lineage commitment and differentiation of pancreatic progenitors, whereas Wnt signaling maintains the stem cell state. The opposing activities of Notch and Wnt have also been found in the skin, mammary glands and intestinal stem cells [135,136,137]. Similarly, both pathways can act on the same cell during the development of sensory bristle and epidermal cells in *Drosophila* and *Xenopus* [138,139]. In recent years, genetic analysis and proposed mathematical models have explained the dynamics of the crosstalk between the Notch and Wnt pathways. Here, we will summarize the major findings concerning the molecular mechanisms of the interactions between the two signaling pathways. First, Dishevelled 2 (Dvl2) plays a dual role, acting as an activator of Wnt signaling and an inhibitor of Notch activity. Dvl2 blocks Notch signaling directly after interacting with the Notch carboxyl terminus, which results in the disruption of the lateral inhibition signal mediated by Notch in the sensory mother cells (SMCs) of *Drosophila* [138] (Figure 5). Collu *et al.* [139], found that Dvl2 binds and inhibits Rbp-J proteins in order to suppress the transcriptional activity of the Rbp-J-NICD-Maml transcriptional activator complex during *Xenopus* epidermal development (Figure 5). The crosstalk mechanism is conserved between vertebrates and invertebrates, as Dvl2 targets the unique and common pathway component, Rbp-J, a core player in conventional Notch signaling. The similar inhibition of Notch signaling by Dvl2 has been shown during the establishment of planar polarity in the *Drosophila* eye and leg epithelium [140,141]. The regulation of Notch signaling by the Wnt pathway is shown by the negative effect of Wnt on the Dvl2-mediated GSK3β activity. GSK3β stabilizes the Notch-IC by binding and phosphorylating Notch-IC in the embryonic fibroblasts and N2a cells [142], and GSK-3β inhibition leads to the degradation of Notch-IC mediated by the proteasome (Figure 5). Furthermore, Notch can directly interact with β-catenin. Notch regulates the stability and activity of Armadillo/β-catenin and negatively regulates β-catenin/TCF transcription in *Drosophila* [143]. In neural precursor cells and vascular progenitors, NICD inhibits β-catenin activity directly by forming a Notch/β-catenin/Rbp-J complex to prevent β-catenin from binding its target sites [144]. The protein complex may play a critical role in cell fate determination in various organs. Lastly, Wnt signaling inhibits Notch activity through Pygopus2 (Pygo2) to promote self-renewal and to prevent the premature differentiation of mammary stem cells (MaSCs) [145]. Pygo2, a histone methylation reader and a context-dependent Wnt/β-catenin coactivator, facilitates the binding of β-catenin to the Notch3 locus and maintains Notch3 in a bivalent chromatin structure in MaSCs [145]. The crosstalk between Wnt and Notch reinforces the balance among stem cells, progenitors and differentiated cells within a tissue, and these confirmed molecular interactions might occur in the pancreas.

### 5.2. Notch/FGF Crosstalk

**Figure 6:**
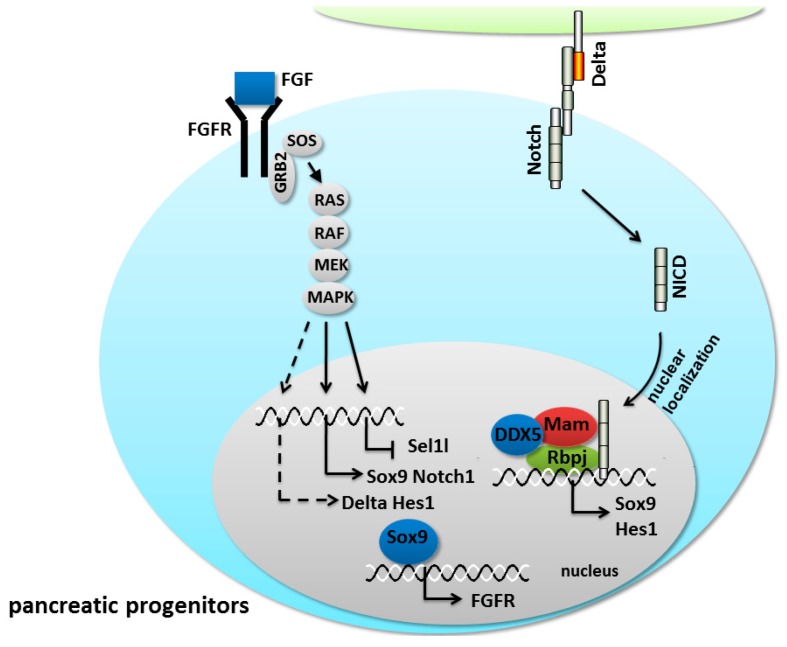
The crosstalk between the fibroblast growth factor (FGF) and Notch pathways in pancreatic progenitors. The dashed lines signify that the mechanisms existing in the pancreas are unknown. The arrows represent activation whereas bar-headed lines represent inhibition.

FGF signaling from the pancreatic mesenchyme has been shown to play an essential role in pancreatic development and the pancreatic disease process. Studies using recombined models of embryonic tissues have shown that pancreatic buds can develop *in vitro* but will not undergo any growth or branching morphogenesis without the presence of the mesenchyme [146,147]. FGF10 signaling plays a crucial mitogenic role in driving the proliferation of pancreatic progenitor cells [64,148,149]. In *FGF10^−/−^* mouse embryos, the proliferation of Pdx1^+^ progenitor cells was reduced, but the growth, differentiation and branching morphogenesis of the pancreatic epithelia were arrested [148]. In mice, the persistent expression of *FGF10* mediated by the Ipf1/Pdx1 promoter increased the proliferation of pancreatic progenitor cells and arrested them in a pluripotent state [110]. Furthermore, the expression of Notch1 and Hes1 was maintained in the pancreatic epithelium, along with the reduction of Ngn3 [110]. The persistent expression of *FGF10* perturbs the expression of Suppressor enhancer lin12/Notch 1-like (Sel1l), which regulates pancreatic epithelial growth and differentiation by suppressing Notch signaling in mice [110,150]. The phenotype of mice with an overexpression of *FGF10* is similar to that of the mice with Notch overexpression described above. Thus, Notch signaling is a critical downstream effector of FGF pathway-induced embryonic pancreatic epithelial proliferation (Figure 6). FGF-stimulated progenitor cell maintenance via Notch signaling has been previously reported in several developmental contexts. During tracheal cell invagination in *Drosophila*, the FGF-like ligand Branchless activates the FGF-receptor, and the downstream MAPK signaling causes the upregulation of Δ [151]. Likewise, FGF10 is capable of maintaining the dental epithelial precursor pool via the stimulation of Hes1 in Fringe-dependent or Fringe-independent manners in the developing tooth [152]. However, whether the above mechanisms exist in the pancreas is unknown (Figure 6). Recently, mouse genetic studies have revealed that there is a FGF10/FGFR2b/Sox9 feed-forward loop in early pancreatic progenitors to maintain their proliferation [31] (Figure 6). Thus, Sox9 acts as the conduit between FGF10 and Notch in the proliferation of progenitors.

Classic culture explant experiments highlight the importance of the mesenchyme for exocrine pancreatic growth and differentiation [153,154]. In *Pdx1-FGF4* transgenic mice, the pancreas has degenerated ductal and destructive endocrine tissue [155]. However, the exact function of FGF signaling on β-cell development remains controversial. For example, studies on the *FGF10* knockout mice have suggested that FGF10 could directly and positively control the final number of β-cells [148,156,157]. Mice with attenuated FGFR1c have a decreased number of β-cells and develop diabetes. [158]. On the other hand, the overexpression of FGF10 in the pancreas inhibited endocrine fate by increasing Notch signaling in both mice and rats [110,159].

## 6. Conclusions and Future Perspective

The Notch pathway regulates cell fate and homeostasis during the development and postnatal life of self-renewing tissues. It not only plays important roles in the pancreas but also regulates self-renewal, lineage specification and differentiation of stem cells in other systems. Notch signaling functions at different time and at different levels during the development of the central nervous system (CNS). Initially, the Notch pathway enhances the neural precursor proliferation and represses their differentiation [160,161]. At later stages, Notch signaling promotes astrocyte differentiation and inhibits oligodendrocyte generation [162]. Notch signaling maintains self-renewal of the early muscle progenitors and regulates their differentiation during embryonic development and adulthood [163,164]. Studies with flies, nematodes, and vertebrates have revealed that Notch signaling is an evolutionarily conserved mechanism and specifies cell fates through local cell interactions in nearly all tissues [165,166,167]. However, there may be species- and tissue-specific differences in the precise roles of the Notch pathway in regulating stem cells and their fate. Notch signaling is an important player that operates sequentially and spatially to affect different aspects of pancreas formation. During early pancreatic development, distinct levels of Notch signaling at two different transition stages trigger the proliferation and differentiation of progenitors; strict control of the time and dosage of the Notch signaling components is necessary for proper organ homeostasis. We speculate that the Notch-mediated regulation of endocrine or exocrine differentiation may be more complex than we currently understand.
